# Case of Mollities Ossium, Preceded by Degeneration of the Muscles

**Published:** 1854-09

**Authors:** Thomas K. Chambers

**Affiliations:** Physician to St. Mary’s Hospital


From the London Lancet.

*Case of Mollities Ossium, preceded by Degeneration of the Muscles.* By THOMAS K. CHAMBERS, Physician to St. Mary's Hospital.

The case was that of a young woman, twenty-six years of age, admitted in St. Mary's Hospital in March, 1853. She had never been able to follow any calling, on account of weak health. The principal features of the case, in the early stage, consisted in defective muscular power, the flesh of the body feeling exceedingly soft and flabby, the calf hanging down flaccid and baggy. During her residence at St. Mary's, the bones of the back and limbs were examined several times, without any deviation from the natural state being discovered. Spontaneous fracture, first of one femur and afterwards of the other, occurred at St. George's Hospital; and subsequently very remarkable changes in the osseous structures took place. Thus, in April, 1853, the right arm became painful to the touch, and paralytic. In May the same misfortune happened to the left upper extremity; in June the pelvic arch gave way; in July the ribs on the right side fell in, and she began to suffer much from dyspnœa and cough; in August the bones of both arms were quite soft. Towards the end of October the distortion of the lower parts of the trunk was so great, that the feces could not naturally be expelled. She died in November. The bones throughout the whole system were found soft and unresisting, and a sharp instrument could be readily passed through them. A section of the tibia was of the color of muscle, and presented to the knife scarcely more resistance than brain, its shape being retained by the aid of the tough periosteum. The microscope exhibited the bone as consisting of large fat vesicles, some containing a white, others a reddish oil. The parts next the periosteum, which felt gritty, presented, when examined under a quarter inch glass, small islands of opaque bone, the bone-corpuscles being indistinct, and the canaliculi not to be discovered. The muscular fibre presented everywhere the characteristics of granular degeneration. The account concludes by an enumeration of the points of the case most worthy of attention :—

1. The portrait which was afforded at an early stage of the disease—a stage at which it was rarely the subject of observation.

2. The impression produced by it—viz., that the degeneration of the bones was preceded by that of the muscles, and that the degeneration of the two tissues was dependent on the same crisis; and the probability, therefore, was, that such was the history of analogous cases.

3. The fact that the degeneration was least advanced in the external circumference of the bone.

4. The formation of perfect fat vesicles in both bone and muscles.

